# AccuCalc: A Python Package for Accuracy Calculation in GWAS

**DOI:** 10.3390/genes14010123

**Published:** 2023-01-01

**Authors:** Jana Biová, Nicholas Dietz, Yen On Chan, Trupti Joshi, Kristin Bilyeu, Mária Škrabišová

**Affiliations:** 1 Department of Biochemistry, Faculty of Science, Palacký University in Olomouc, 78371 Olomouc, Czech Republic; 2 Division of Plant Sciences, University of Missouri, Columbia, MO 65201, USA; 3 Christopher S. Bond Life Sciences Center, University of Missouri, Columbia, MO 65212, USA; 4 MU Data Science and Informatics Institute, University of Missouri, Columbia, MO 65212, USA; 5 Department of Electrical Engineering and Computer Science, University of Missouri, Columbia, MO 65212, USA; 6 Department of Health Management and Informatics, School of Medicine, University of Missouri, Columbia, MO 65212, USA; 7 Plant Genetics Research Unit, United States Department of Agriculture-Agricultural Research Service, University of Missouri, Columbia, MO 65211, USA

**Keywords:** python package, GWAS, accuracy, causative mutation, SP2CM, Manhattan plot

## Abstract

The genome-wide association study (GWAS) is a popular genomic approach that identifies genomic regions associated with a phenotype and, thus, aims to discover causative mutations (CM) in the genes underlying the phenotype. However, GWAS discoveries are limited by many factors and typically identify associated genomic regions without the further ability to compare the viability of candidate genes and actual CMs. Therefore, the current methodology is limited to CM identification. In our recent work, we presented a novel approach to an empowered “GWAS to Genes” strategy that we named Synthetic phenotype to causative mutation (SP2CM). We established this strategy to identify CMs in soybean genes and developed a web-based tool for accuracy calculation (AccuTool) for a reference panel of soybean accessions. Here, we describe our further development of the tool that extends its utilization for other species and named it AccuCalc. We enhanced the tool for the analysis of datasets with a low-frequency distribution of a rare phenotype by automated formatting of a synthetic phenotype and added another accuracy-based GWAS evaluation criterion to the accuracy calculation. We designed AccuCalc as a Python package for GWAS data analysis for any user-defined species-independent variant calling format (vcf) or HapMap format (hmp) as input data. AccuCalc saves analysis outputs in user-friendly tab-delimited formats and also offers visualization of the GWAS results as Manhattan plots accentuated by accuracy. Under the hood of Python, AccuCalc is publicly available and, thus, can be used conveniently for the SP2CM strategy utilization for every species.

## 1. Introduction

Genome-wide association study (GWAS) is a widely-used statistical method for finding associations between phenotype and genotype. The analysis fits the data into one of the mathematical models (typically the linear regression model) and discovers genomic regions linked with a phenotype. Within GWAS, the *p*-value is traditionally calculated for every genetic variant present in the genotype data as a statistical measure of an association with a phenotype [1,2]. Due to linkage disequilibrium, the associated genetic variants usually form regions [3]. These associated regions can be the first step in discovering the phenotype marker or causal gene responsible for the observed phenotype [1]. Nevertheless, the real meaning of the *p*-value is the probability of seeing the observed data or even more extreme data in the case that the null hypothesis (H0) is true. The typical H0 is that the experimental genetic variant does not affect the observed trait. Thus, the contribution to the model is none. For the alternative hypothesis, H1, the observed genetic variant affects the observed trait; thus, it contributes to the model. The lower *p*-value than a set threshold is then taken as an indication of the H1 validity [4,5]. However, even though GWAS successfully identified causative mutations (CMs) [6,7], the genetic variant with the lowest *p*-value is not always the most highly-associated variant with phenotype [8]. This is due to the following complicating factors: fitting models (only a simplified version of the actual situation), genetic complexity of traits, the density of genotype data, the distribution of phenotypes, or multiallelic origins of CMs. Thus, GWAS serves only as a mathematical approximation of the relationship between genotype and phenotype that can be far from perfect [8].

There are more types of genotype data used for GWAS, but generally, we can classify them into one of two types: genotyping data and resequenced data. Genotyping data are of low density and thus cover only a small percentage of the whole genetic variation that is represented only by a subset of the SNPs. Obtaining genotyping data is easier, faster, and less costly than in the case of resequenced data. These advantages allow the analysis of a larger number of samples within a single study. However, genotyping-data-based GWAS discoveries rarely lead to direct CM identification. In comparison to genotyping, resequencing can reveal the majority of total genetic variation if a sufficient number of diverse samples is present in a dataset and resequencing quality parameters and depth are high. Nevertheless, obtaining sufficient amounts of high-quality resequenced genotype data for a larger number of samples is still limited by its cost and tedious process of data generation, processing, and curation. Therefore, GWAS analysis of larger resequenced datasets is less affordable, thus, limiting CM identification [3,4,9,10,11].

In our previous work [12], we developed a new strategy for leveraging both low-density genotype and resequenced data for CM discovery through the “Synthetic phenotype association study” (SPAS). This approach subsidizes missing phenotype information of publicly available resequenced soybean datasets by a high-accuracy, low-density genotype marker that is used as a synthetic phenotype in GWAS. Whilst SPAS reveals the landscape of association for genomic variants and phenotypes; accuracy serves as a measure of direct correspondence between every variant position and phenotype. Therefore, SPAS, empowered by accuracy calculation, extends the analysis beyond a threshold of statistical significance of a fitting model used for GWAS, limits the number of false negatives [12], and thus overcomes some of the GWAS limitations [8]. For soybean, we introduced a web-based AccuTool for a reference diversity panel of resequenced soybean accessions (Soy775) that automatically calculates the accuracy for 35.7 M curated variant positions [12] that are publicly available via SoyKB [13]. To extend this accuracy calculation for other soybean genotype datasets with more or different variant positions, to make accuracy calculation more effective for disproportional phenotype datasets, and, also, to enable SP2CM for other species, we developed AccuCalc as a Python-based package. In comparison to AccuTool, the AccuCalc package presented in this work contains all previously described functions and options related to accuracy calculation and, further, is empowered by additional accuracy types that are more suitable for disproportional data. AccuCalc is enriched by synthetic phenotype formatting from user-provided genotype and genome-localized association study (GLAS) visualization. AccuCalc is GWAS fitting model-independent since it uses GWAS results provided by a user as input. The AccuCalc package design accepts either variant calling format (vcf) or HapMap (hmp) genotype format and can be used for any species. AccuCalc offers the ability to extend the use of whole genome sequence data that are becoming more available from previous and new studies [13,14,15].

Our previously developed AccuTool is a web-based tool that allows users to interact with its web interfaces to perform an analysis on one soybean dataset. The performance of AccuTool is bounded by the hosting machine/services, the R-shiny framework, and stable internet connections for which AccuTool necessitates being online and publicly accessible. AccuTool needs the hosting machine’s random-access memory (RAM) and storage space to function properly. The R-shiny framework limits the data upload size, which can negatively impact the user experience. AccuCalc, on the other hand, is a package developed using the Python programming language, and users can interact with scripts within the package in the console/terminal to perform analysis. With this approach, users not only do not need to rely on the R-shiny framework but also have more control over the console/terminal. AccuCalc can be run on any Linux or Windows machine that has enough RAM and disk space. Data that feeds into AccuCalc also do not need to go through a time-intensive data upload process. Upon completion of data analysis, users can also choose the data to keep or transfer to any other data-storing infrastructures or services.

In this work, we document the development and utilization of AccuCalc. Further, we present two examples of AccuCalc utilization: A case study of a high-accuracy tagging marker identification for soybean flower color as an example of Synthetic phenotype to causative mutation (SP2CM) Part1 accuracy-based identification with proportional distribution of phenotypes and a case study of CM identification for a rare phenotype of soybean trichome loss, known as a glabrous phenotype for the pubescence density trait.

## 2. Materials and Methods

The AccuCalc design is based on the analytic approach previously published by our group [12]. The genetic variants with significant *p*-value calculated in GWAS for the trait of interest are evaluated by the accuracy calculation that reflects the direct correspondence to selected phenotypes arranged into a binary phenotype. The accuracy calculation is computed in four distinct ways to cover more types of data structure. The average accuracy realistic (Avr_acc) is calculated according to Equation (1):

(1) $$\mathrm{Avr}\_\mathrm{acc}=\frac{\mathrm{Acc}\_\mathrm{MUT}+\mathrm{Acc}\_\mathrm{WT}}{2}\times 100$$

A strict measure of direct correspondence between known phenotype and genotype values of accessions with matching WT and MUT phenotypes to genotype, where WT accuracy (Acc_WT) and MUT accuracy (Acc_MUT) are given by the Equations (2) and (3). N_WT-REF_ represents the count of variants with WT phenotype and Ref allele, and N_WT-ALT_ represents the count of variants with WT phenotype and Alt allele. Similarly, N_MUT-ALT_ represents the count of variants with MUT phenotype and Alt allele, and N_MUT-REF_ represents the count of variants with MUT phenotype and Ref allele:

(2) $$\mathrm{Acc}\_\mathrm{WT}\;=\;\frac{N_{\mathrm{WT}-\mathrm{REF}}}{N_{\mathrm{WT}-\mathrm{REF}}+{\;N}_{\mathrm{WT}-\mathrm{ALT}}}\times 100$$

(3) $$\mathrm{Acc}\_\mathrm{MUT}\;=\;\frac{N_{\mathrm{MUT}-\mathrm{ALT}}}{N_{\mathrm{MUT}-\mathrm{REF}}+{\;N}_{\mathrm{MUT}-\mathrm{ALT}}}\times 100\;$$

Combined accuracy (Comb_acc_real) summarizes only the available phenotype and genotype data for every variant and ignores the sample distribution and missing information, according to Equation (4). The variables in the equation are the same as in the case of Equations (2) and (3):

(4) $$\mathrm{Comb}\_\mathrm{acc}\_\mathrm{real}=\frac{N_{\mathrm{WT}-\mathrm{REF}}+{\;N}_{\mathrm{MUT}-\mathrm{ALT}}}{N_{\mathrm{WT}-\mathrm{REF}}+{\;N}_{\mathrm{WT}-\mathrm{ALT}}+{\;N}_{\mathrm{MUT}-\mathrm{REF}}+{\;N}_{\mathrm{MUT}-\mathrm{ALT}}}\times 100$$

The optimistic mode of combined accuracy calculation assumes that the structure and distribution of information in missing data are the same as in the known data. However, the optimistic assumption did not prove to always be a significant measure according to our previous work [12]. The average accuracy gives a better evaluation of the dataset with a strongly disproportional phenotype.

The factor of missing information is included in the average accuracy pessimistic (Avr_acc_pes) calculated according to Equation (5):

(5) $$\mathrm{Avr}\_\mathrm{acc}\_\mathrm{pes}=\frac{\mathrm{Acc}\_\mathrm{pes}\_\mathrm{MUT}\;+\;\mathrm{Acc}\_\mathrm{pes}\_\mathrm{WT}}{2}\times 100$$

where WT accuracy pessimistic (Acc_pes_WT) and MUT accuracy pessimistic (Acc_pess_MUT) are given by Equations (6) and (7). N_WT-REF_ represents the count of variants with WT phenotype and Ref allele, and N_WT-ALT_ represents the count of variants with WT phenotype and Alt allele. Similarly, N_MUT-ALT_ represents the count of variants with MUT phenotype and Alt allele, and N_MUT-REF_ represents the count of variants with MUT phenotype and Ref allele. N_MUT_ represents the count of all variants with the MUT phenotype, and N_WT_ represents the count of all variants with the WT phenotype.

(6) $$\mathrm{Acc}\_\mathrm{pes}\_\mathrm{WT}=\frac{N_{\mathrm{WT}-\mathrm{REF}}}{N_{\mathrm{WT}}}\times 100$$

(7) $$\mathrm{Acc}\_\mathrm{pes}\_\mathrm{MUT}=\frac{N_{\mathrm{MUT}-\mathrm{ALT}}}{N_{\mathrm{MUT}}}\times 100$$

The combined accuracy pessimistic (Comb_acc_pes) is calculated according to Equation (8):

(8) $$\mathrm{Comb}\_\mathrm{acc}\_\mathrm{pes}=\frac{N_{\mathrm{WT}-\mathrm{REF}}+N_{\mathrm{MUT}-\mathrm{ALT}}}{N_{\mathrm{ALL}}}\times 100$$

**Figure 1:**
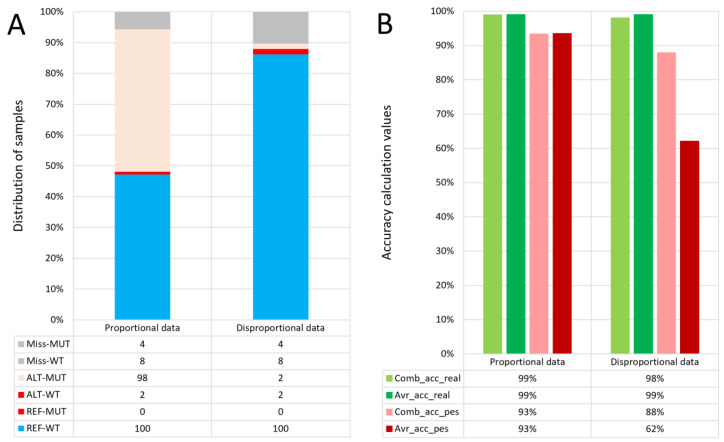
A comparison of proportional and disproportional data based on the accuracies. The figure shows a comparison of two types of datasets with proportional and disproportional data by phenotype. The disproportional data simulates the cases with only a small number of samples with mutant phenotypes available. Plot (**A**) shows the sample distribution transformed into percentages from sample counts. The counts are available in the table below plot (**A**). They represent the number of samples in groups defined by a phenotype and genotype pair. Plot (**B**) shows the difference in four types of accuracy calculations (values available in the table below the plot) according to the dataset type. The accuracy calculations are counted from sample counts shown in plot (**A**).

In this work, we tested that this type of accuracy is an ideal evaluation criterion, especially for disproportional datasets. The difference in the equations for pessimistic calculation is the same as in the case of the optimistic variant. The pessimistic accuracy calculations serve as a simple measurement of the missing information in the selected area, as results with a high amount of missing information may not be as reliable. The combined accuracy calculation can serve as an overview of all samples at a variant position. However, in the case of a disproportional dataset (for example, a lot of controls and only a few cases), the more numerous sample type can skew the combined accuracy. In that case, when there is missing sample data in the selected region in the low-frequency phenotype, it can cause the reliability of the results to drop dramatically because of an insufficient number of samples (Figure 1).

The synthetic phenotype formatting was added to AccuCalc as an option that allows users to re-use resequenced datasets for localizing candidate CMs based on a low-density genotype marker. The AccuCalc procedure is as follows: First, the GWAS results are refined to identify a top associated marker from the variants with significant *p*-values using an accuracy calculation between the phenotype and the significant markers. Second, a position of the highest accuracy/−log10(*p*-value) is formatted as the synthetic phenotype and subsequently used for a further accuracy calculation of variant positions of a resequenced dataset with the aim to identify a CM based on the direct correspondence given by the highest accuracy values for the synthetic phenotype. The synthetic phenotype can be used as a phenotype for studies with the new expanded dataset even without known real phenotype information for samples in the datasets.

Beyond the main function for accuracy calculation and synthetic phenotype creation, the AccuCalc package includes other functions. These auxiliary functions help users to upload, process, analyze, and plot their data seamlessly.

## 3. Results

**Figure 2:**
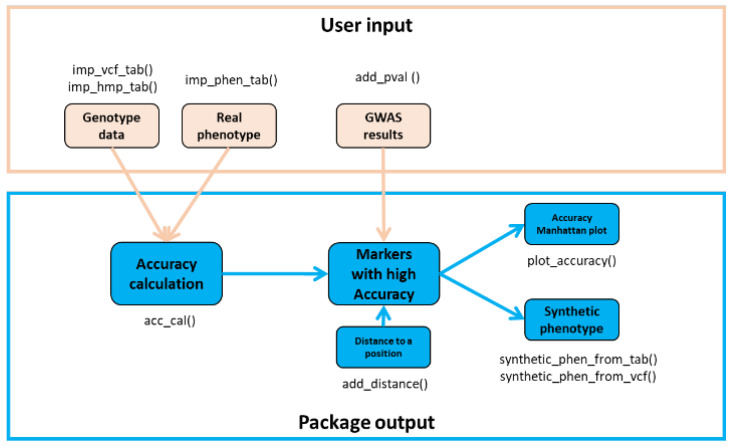
The AccuCalc workflow. Inputs (orange), outputs (blue), and AccuCalc follow the same logic as published in our former work [12].

The AccuCalc package was created and tested with Python (https://www.python.org/, accessed on 27 December 2022), version 3.7.4., and hosted at https://github.com/Biovja/AccuCalc, accessed on 27 December 2022. The package requires the following Python packages and libraries: pandas (https://pandas.pydata.org/, accessed on 27 December 2022, tested with ver. 1.3.3), NumPy (https://numpy.org/, accessed on 27 December 2022) and Matplotlib (https://matplotlib.org/, accessed on 27 December 2022, tested with ver. 3.2.2). For the opening of compressed Variant Call Format files (vcf) (vcf.gz or vcf.bgz) the gzip (https://docs.python.org/3/package/gzip.html, accessed on 27 December 2022, tested with ver. 1.9.0) package was tested and is used in the usage example. The AccuCalc package was tested in environments of both Linux and Windows operation systems and is developed by using stable and widely used Python packages. The package diagram (Figure 2) shows the main functions and their connections.

### 3.1. Accuracy Calculation

One of the main points in the analytic approach covered in AccuCalc is the accuracy calculation. The accuracy calculation is performed by a function called acc_cal(). It takes two compulsory arguments. The first one is called gen_tab and represents an object with genotype information in a proper data format. The second is called phen_tab, which represents an object with phenotype information in proper data format. The optional arguments manage the accuracy calculation settings. The “cal” parameter enables the user to set the phenotype value (Wild type or Mutant) for a reference genome. The “flip” parameter enables the automatic setting of the reference genome according to the best average accuracy. The function generates an Acc_tab. Similarly, as the function input, the Acc_tab is a pandas.DataFrame-based object. The first six columns contain information about individual loci (“#CHROM”, ”POS”, ”ID”, ”REF”, ”ALT”, ”INFO”), and the subsequent columns contain the results of accuracy calculations and additional statistic information for the loci.

### 3.2. Synthetic Phenotype

The second key part of the model analytic approach for AccuCalc is the synthetic phenotype. The synthetic phenotype is provided by the synthetic_phen_from_tab() or synthetic_phen_from_vcf() functions. Both functions generate a pandas.DataFrame-based phen_tab object. The synthetic_phen_from_tab() function serves to create the synthetic phenotype from the existing gen_tab, which is the first mandatory argument of the function. The synthetic_phen_from_vcf() serves to create the synthetic phenotype from the vcf file with the file name and path as the first mandatory argument. The other mandatory arguments in both functions specify the chosen locus and the synthetic phenotype.

The AccuCalc package includes an auxiliary function export_phen_tab(). This function exports the synthetic phenotype as a simple tab-divided text file with the first row as the header and two columns. The first contains the sample’s names, and the second their synthetic phenotype. The mandatory arguments are the phen_tab representing the synthetic phenotype and the string name representing the result file name (the .txt suffix is added automatically).

### 3.3. Import

For importing data into a format suitable for the operations described above the imp_vcf_tab(), imp_hmp_tab(), imp_phen_tab(), and add_pval () functions are contained in the AccuCalc package.

The imp_vcf_tab()/imp_hmp_tab() function creates a simple pandas data frame object gen_tab with a similar structure as the traditional Variant Call Format (vcf) file from a vcf file or hapmap file (viz GAPIT manual). Thus, the first nine columns provide information describing each genetic variant (“#CHROM”, ”POS”, ”ID”, ”REF”, ”ALT”, ”QUAL”, ”FILTER”, ”INFO”, ”FORMAT”) followed by genotype calls for each sample. In gen_tab, the reference alleles are represented by numerical value 0, the alternative alleles are represented by numerical value 1, and the missing or not included in analysis alleles are represented by value −2. This simple format build commonly used in Python object pandas.DataFrame is advantageous for two main reasons. It allows the calculation of the accuracy calculation in a simple way using the NumPy Python package, and it is straightforward for a user to transform their own data into this widely known object in an accessible format. This may be of interest to the user if their genomic data are of a different format type than the vcf file format.

The imp_phen_tab() function creates a simple pandas.DataFrame object phen_tab. The columns represent individual samples, and the only existing row contains the sample phenotype/trait value. As the accuracy calculation is programmed for binary phenotype, phen_tab keeps the phenotype/trait as a simple numerical value. The Wild-type (WT) phenotype is represented by 0, the Mutant type (MUT) is represented by 1, and the missing or not included in analysis phenotypes are represented by −2.

The minor additional function add_pval () adds *p*-values for individual loci from GWAS results values into an existing accu_tab. The function takes two compulsory arguments: acc_tab and pval_file. The acc_tab argument represents a pandas.DataFrame object created during the accuracy calculation by the acc_cal() function. The pval_file argument represents the text or comma-separated values (CSV) file with the results from GWAS analysis for the genome region of interest. The optional arguments serve to specify the format of the GWAS results file as the files differ according to the chosen GWAS tool.

The second auxiliary function is sort_names(). The function sorts the phen_tab according to the order of samples in gen_tab. If the sample from geno_tab is not included in phen_tab, NA is added.

### 3.4. Plotting

The addition of the *p*-values into Accu_tab is a necessary step before plot creation. The function plot_accuracy() generates standardized plots for data in Accu_tab object, which is the only compulsory argument for the function.

### 3.5. Example Cases

**Figure 3:**
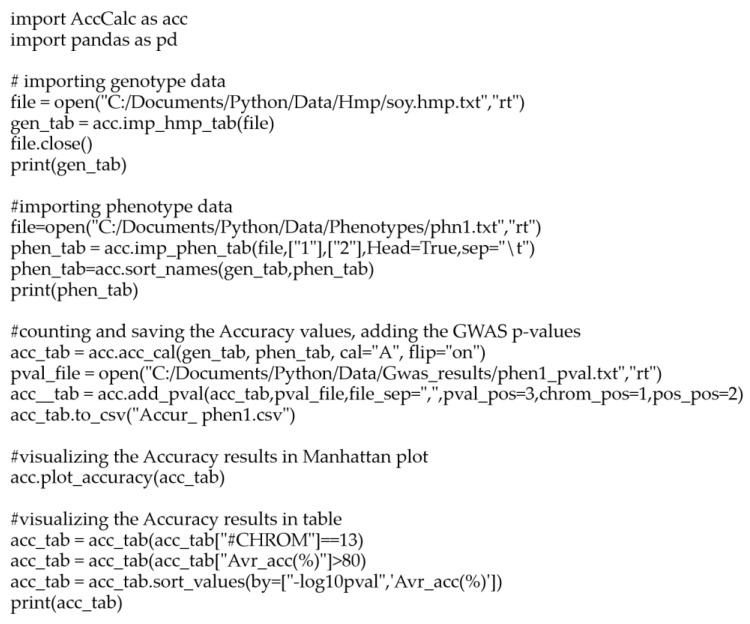
Example of the AccuCalc script. The AccuCalc script section describes the accuracy calculation and accentuation of the Manhattan plot with average accuracy for the case study of the flower color-associated region on chromosome 13. The option to filter based on average accuracy values >80% is shown.

The usage of the AccuCalc package requires basic Python. From importing the data to its processing and obtaining the results, only a few common Python commands, together with the AccuCalc function, are needed, as demonstrated in the package usage example script (Figure 3).

The script shows the usage of the package from importing the genotype and phenotype data, counting their accuracy, and saving the results. The results are presented in the form of a text table (in a csv file) and a predefined Manhattan plot (both genome-wise and chromosome-wise variants) supplemented with the accuracy information visualized with a color scale.

#### 3.5.1. Case Study 1: High-Accuracy Tagging Marker Identification

**Figure 4:**
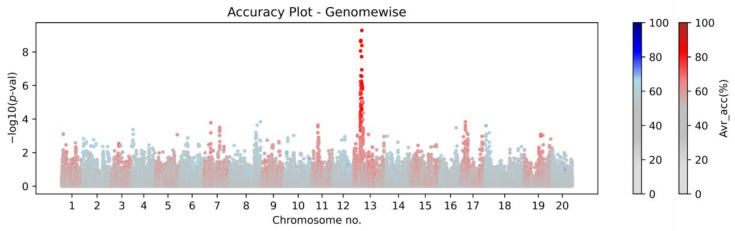
The AccuCalc genome-wise view of genome-wide association study data accentuated with calculated accuracy. The Manhattan plot for soybean data (Illumina SoySNP50K DNA bead chip genotyping data and purple/white flower color phenotype data). The Manhattan plot is accentuated with the average accuracy of the causative mutation-associated SNP on chromosome 13 at position 17,316,756: even chromosomes (blue-gray scale), odd chromosomes (red-gray scale). AccuCalc enables the accuracy Manhattan plot creation and provides a table with the accuracy calculation and summary information for every included variant in the analysis.

**Figure 5:**
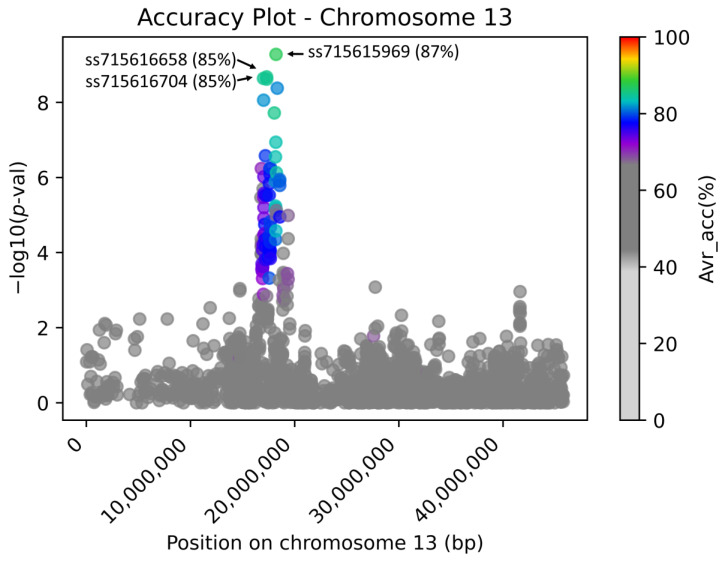
The AccuCalc chromosome-wise view of the associated region with flower color on chromosome 13 accentuated with calculated accuracy. The Manhattan plot for soybean data (SoySNP50K chip genomic data and purple/white flower color phenotype data) with average accuracy to the causative mutation-associated SNP on chromosome 13 at position 17,316,756. In this example, the markers with the highest accuracy (shown for the three top points in parentheses) are also the markers with the highest −log10 (*p*-values).

The first key decision that is required for the SP2CM strategy is to select the most accurate, highly associated tagging marker (SP2CM-Part 1). In this work, this step is demonstrated on soybean, but the approach was also tested on other organisms (rice, cotton, and Arabidopsis; non-public data and not part of this publication). We used the previously described flower color trait as an example of a simple qualitative binary phenotype with proportional distribution, where the disturbed biosynthesis of purple pigments in flowers causes white flower color [16]. In this work, we utilized AccuCalc to assess the accuracy of the associated low-density markers in predicting purple or white flower colors on a test dataset (42 accessions with WT purple flowers, 98 accessions with MUT white flowers, and 7 with unknown NA phenotypes are publicly available at https://soykb.org/, accessed on 27 December 2022 and https://soybase.org/, accessed on 27 December 2022). This phenotype underlies the *W1* gene [17] on chromosome 13, where a SNP at position 17,316,756 (*Glyma.13.072100*—Williams 82.a2v1) is associated with the causative Indel [12,18]. For genotype data, we used low-density Illumina SoySNP50K DNA bead chip [19] genotyping data that are publicly available at USDA Soybean Germplasm Collection (GRIN, Urbana, IL, USA), and accuracy to the CM associated SNP, as described in our previous work [12]. Figure 4 documents the genome-wide Manhattan plot with a detected *W1*-associated region on chromosome 13, and Figure 5 illustrates the zoomed *W1* region with accentuated Avr_acc to the real flower color phenotype (the AccuCalc output file is available at https://github.com/Biovj/AccuCalc/tree/main/publication_files, accessed on 27 December 2022).

#### 3.5.2. Case Study 2: CM Identification for a Rare Phenotype

**Figure 6:**
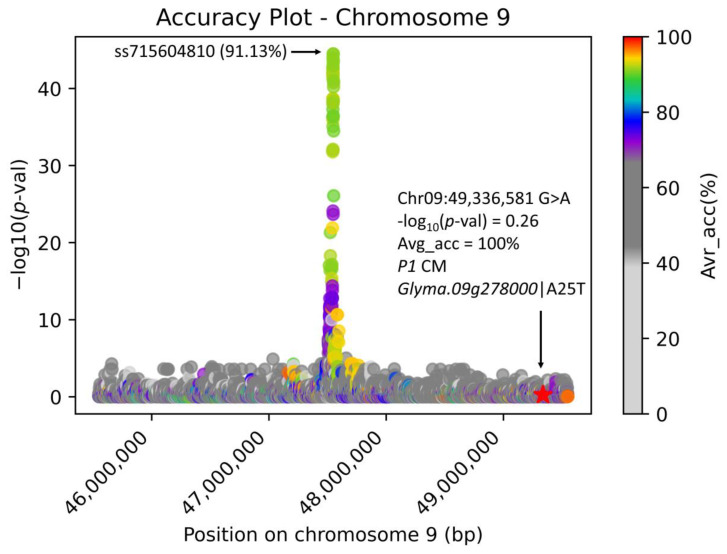
The AccuCalc chromosome-wise view of the 4 M bp region on chromosome 09 with the associated region to ss715604810, *P1* tagging marker [16]. The Manhattan plot for the soybean USB15x(302) resequenced data and ss715604810 synthetic phenotype with Avr_accu of the glabrous/pubescent phenotype. The arrow points to the *Glyma.09g278000*-A25T causative mutation.

Here, we demonstrate that AccuCalc can identify a CM for a very rare phenotype without the need to resequence additional accessions. Pubescence density is controlled by many alleles, where *P1/p1* was confirmed to be *Glyma.09g278000* (Williams 82.a2.v1), where a missense CM A25T determines the complete loss of pubescence, known as the glabrous phenotype [20]. In our previous work, we used the USB15x(302) subset of USB481-resequenced accessions [21] as a model dataset. In this dataset, there is a single glabrous accession (USB-251 PI 548178) with the missense CM A25T on chromosome 09 caused by SNP G>A at position 49,336,581 Williams 82.a2.v1. Therefore, we used this dataset as an example of extremely disproportional data where GWAS cannot be performed. Here we used a previously identified tagging marker (SoySNP50K DNA bead chip) for glabrous versus normal pubescence density ss715604810 (chr09: 47,548,832 T>C) [16] as a synthetic phenotype (241 REF allele T, 53 MUT C, 6 NA) in GLAS) of SP2CM-Part 2 [12] on a 4 M bp genomic region (centered on the ss715604810 marker) and calculated the accuracy for glabrous (*n* = 1)/pubescent (*n* = 299) phenotypes (the AccuCalc output file is available at https://github.com/Biovj/AccuCalc/tree/main/publication_files, accessed on 27 December 2022). Figure 6 documents the ss715604810-associated region with accentuated Avr_acc to the glabrous/pubescent phenotype with the position of the *P1* CM highlighted. There are 50,912 total variant positions in the analyzed 4M bp genomic region of the USB15x(302) on chromosome 09, where only 12 variant positions possess the highest (100%) Avr_acc (Table 1) with varying pessimistic accuracies. Here, among the 12 highest Avr_acc variant positions, only 1 variant position causes an amino acid change in the translated protein and, therefore, is considered the candidate CM with the highest probability. This CM was experimentally proven by constructing loss-of-function mutants [20].

## 4. Discussion

AccuCalc enables the utilization of the SP2CM strategy for various genotype data inputs without the need for non-public data uploads or access to high-computing clusters. In comparison to our previously developed AccuTool [12], AccuCalc works on custom datasets, offers an option to prepare the synthetic phenotype from a user-defined variant position present in the dataset, and calculates accuracy to both real and synthetic phenotypes. AccuCalc produces Manhattan plots with accentuated accuracy and saves results in a user-friendly tabular format for further analysis. We demonstrated two major AccuCalc analyses: 1) identification of high-accuracy tagging markers for SP2CM—Part 1, and 2) SP2CM—Part 2, CM identification.

Regardless of the GWAS fitting model used, AccuCalc calculates the direct correspondence between markers and phenotypes and assists in the identification of the highest accuracy markers for subsequent SP2CM—Part 2. The case study with flower color shows that regardless of the *p*-value obtained by GWAS, among 2684 marker positions on chromosome 13 where the W1 is located, only 18 markers possessed >80% Avr_accu to purple/white flower color with a single marker with the highest Avr_accu value (87.47%). Further, there were 34 marker positions with Avr_acc 80–75%, 2525 marker positions with Avr_accu between 75% and 50%, and only 106 marker positions with 50% Avr_acc. The AccuCalc output file is available at https://github.com/Biovj/AccuCalc/tree/main/publication_files, accessed on 27 December 2022.

The extreme case of the disproportionate distribution of phenotypes in the dataset, for the example of glabrous versus pubescent phenotypes, has shown that AccuCalc can easily identify the CM in cases where GWAS cannot be applied.

The computational cost is indeed variable depending on the computation power available (common PC station versus a high computing cluster). However, since AccuCalc is intended to be executed on a PC, we calculated the running time for accuracy analysis and plotting of genome-wide GWAS data for SoySNP50K DNA bead chip low-density genotype data for the flower color case study for a common Intel(R) Core(TM) i7-1065G7 CPU @ 1.30GHz 1.50 GHz processor, RAM 16.0 GB, on a Windows ×64 platform. The process was successfully completed in 2 min.

The AccuCalc package helps users process the GWAS results in conjunction with binary or binned phenotypes. The first challenge for users may be the proper categorization of the phenotype into a binary format of two bins in the context of the knowledge of the genomic complexity of the trait. Within our future tool development, we would like to focus on accessible phenotype categorization.

## 5. Conclusions

We have developed a new Python package called AccuCalc for the evaluation of GWAS results. The package provides functions for mathematical evaluation through accuracy calculation for GWAS results for binary and binned phenotypes. It also includes functions for synthetic phenotype formatting, a way to leverage both resequenced datasets with missing phenotype info and genotyping data limited by the number of variant positions on a chip. AccuCalc is publicly available at https://github.com/Biovja/AccuCalc, accessed on 27 December 2022.

## Figures and Tables

**Table 1:** Selection of the highest Avr_accu (100%) variant positions to the glabrous/pubescent phenotype in the 4M bp genomic region on chromosome 09. The table was derived from the AccuCalc analysis output (at https://github.com/Biovj/AccuCalc/tree/main/publication_files, accessed on 27 December 2022) and simplified. The causative mutation position of *Glyma.09g278000*-A25T is in bold.

Chr	Position	Distance to ss715604810 (bp)	REF	ALT	Gene ID	Effect	−log10(p)	Avr_acc (%)	Avr_acc_pes (%)	Acc_pes_WT (%)	Comb_acc_real (%)	Comb_acc_pes (%)	Missing_WT_gen (%)	Count WT	Count MUT
9	46,340,547	1,208,285	A	G, C	*Glyma.09g241100*	intron_variant	0.26	100	99.33	98.66	100	98.67	1.34	299	1
9	47,953,315	404,483	G	A	*Glyma.09g261600*	upstream_gene_variant	0.26	100	99.33	98.66	100	98.67	1.34	299	1
9	47,318,232	230,600	G	A	*Glyma.09g253300*	upstream_gene_variant	0.26	100	99.16	98.33	100	98.33	1.67	299	1
9	47,124,449	424,383	C	G	*Glyma.09g250500*	intron_variant	0.26	100	99.16	98.33	100	98.33	1.67	299	1
9	48,649,742	1,100,910	G	C	*Glyma.09g269100*	downstream_gene_variant	0.26	100	99.16	98.33	100	98.33	1.67	299	1
**9**	**49,336,581**	**1,787,749**	**G**	**A**	***Glyma.09g278000***	**missense_variant A25T**	**0.26**	**100**	**99.16**	**98.33**	**100**	**98.33**	**1.67**	**299**	**1**
9	48,879,536	1,330,704	C	T	*Glyma.09g272300*	intron_variant	0.26	100	99.16	98.33	100	98.33	1.67	299	1
9	45,868,448	1,680,384	G	A	.	intergenic_region	0.27	100	98.83	97.66	100	97.67	2.34	299	1
9	46,493,848	1,054,984	A	G	.	intergenic_region	0.22	100	98.16	96.32	100	96.33	3.68	299	1
9	46,863,256	685,576	A	G	*Glyma.09g247100*	upstream_gene_variant	0.01	100	91.64	83.28	100	83.33	16.72	299	1
9	46,897,306	651,526	A	C	*Glyma.09g247500*	intron_variant	0.23	100	90.47	80.94	100	81	19.06	299	1
9	47,196,829	352,003	T	TATGA, TATGTATATGA	*Glyma.09g251500*	intron_variant	0.43	100	89.46	78.93	100	79	21.07	299	1

## Data Availability

The AccuCalc package is available at https://github.com/Biovja/AccuCalc (accessed on 27 December 2022).
